# Healthy Migrants in an Unhealthy City? The Effects of Time on the Health of Migrants Living in Deprived Areas of Glasgow

**DOI:** 10.1007/s12134-016-0497-6

**Published:** 2016-05-07

**Authors:** Ade Kearns, Elise Whitley, Matt Egan, Catherine Tabbner, Carol Tannahill

**Affiliations:** 1 Urban Studies, School of Social and Political Sciences, University of Glasgow, 25 Bute Gardens, G12 8RS Glasgow, UK; 2 CSO/MRC Social and Public Health Sciences Unit, University of Glasgow, Glasglow, UK; 3 London School of Hygiene and Tropical Medicine, London, UK; 4 Glasgow Centre for Population Health, Glasglow, UK

**Keywords:** Healthy migrant effect, Social and economic migrants, Asylum seekers and refugees, Deprived areas, Migrant concentrations

## Abstract

This paper examines the healthy immigrant effect in Glasgow, a post-industrial city where the migrant population has more than doubled in the last decade. Using data from a community survey in 15 communities across the city, the paper compares four health outcomes for the following three groups: British-born, social and economic migrants and asylum seekers and refugees. Migrants were found to be healthier than the indigenous population on all four measures, particularly in the case of adult households in both migrant groups and for older asylum seeker and refugee households. Health declines for social and economic migrants with time spent in the UK, but there is no clear pattern for asylum seekers and refugees. Health declined for refugees according to time spent awaiting a decision, whilst their health improved after a leave-to-remain decision. Indigenous and social and economic migrant health declines with time spent living in a deprived area; this was true for three health indicators for the former and two indicators for the latter. Asylum seekers and refugees who had lived in a deprived area for more than a year had slightly better self-rated health and well-being than recent arrivals. The study’s findings highlight the role of destination city and neighbourhood in the health immigrant effect, raise concerns about the restrictions placed upon asylum seekers and the uncertainty afforded to refugees and suggest that spatial concentration may have advantages for asylum seekers and refugees.

## Introduction

### The Healthy Immigrant Effect

From earlier concerns in Western countries that immigrants either represented a health risk to the host population, or were merely heavy users of health care services (Hyman 2001), there is a growing recognition that immigrants are often healthier than the receiving population. This has been reported for example in respect of Canada (Newbold and Danforth 2003; McDonald and Kennedy 2004), the USA (Antecol and Bedard 2006) and Australia (Biddle et al. 2007). The notion of the healthy immigrant effect has the following two distinct elements: first, that new immigrants are healthier than the indigenous population on arrival and second, that this health advantage deteriorates over time as the health of immigrants converges towards that of the host population (Halli and Anchan 2005; Rechel et al. 2013).

Different explanations have been offered for these two components. For the first part, the confounding effects of age are an issue, with recent immigrants tending to be younger, thus making it difficult to separate the effects of shorter periods of residence from those of relative youthfulness. However, a healthy immigrant effect has also been found amongst older age groups (Gee et al. 2003). Three other kinds of explanation for the initial health advantage of immigrants have been suggested. First, self-selection, whereby those people with the best ‘financial and physical means to migrate’ will do so (Kennedy et al. 2015). Moreover, as the costs of migration rise, selectivity increases, especially by level of skill, and relative costs of those skills (Jasso et al. 2004), by education, and, as a result, also according to health (*ibid.*). Second, immigrants often come from countries with healthier lifestyles and cultures than their Western destinations, so that unhealthy behaviours are less common amongst immigrants (Dunn and Dyck 2000). Third, the destination country screening process uses criteria which favour individuals with healthy characteristics and select those with levels of income and education which are associated with better health and healthier lifestyles. Many Western countries operate with a points system for skilled workers to ensure this kind of outcome from the immigration process. On the other hand, the effectiveness of limited compulsory health screening for migrants has been questioned (Coker 2003). Given all these factors, one would expect that economic migrants would be likely to have a health advantage and that asylum seekers and refugees who are forced to leave their home country, for example due to conflict, would be less likely to exhibit healthier characteristics.

Several explanations have been offered for the second component of the healthy immigrant thesis. First, it is argued that social integration and acculturation occur over time, and part of that process involves the adoption of indigenous beliefs, attitudes and behaviours such as smoking, drinking and diet, as a result of which migrant health declines (Chen et al. 1996; Perez 2002; Lara et al. 2005). Second, immigrants are said to experience stress from living in poverty and poor conditions and from facing harassment and discrimination which results in health deterioration (Noh and Kaspar 2003; Utresky and Mathieson 2007). Thus, we might expect the deterioration of migrant health to be worse for those immigrants living in rented housing in deprived areas of western cities. Third, structural barriers to good health and health care are identified as contributors to poor health over time, including poor housing conditions, low income and education, lack of jobs and poor language skills; these may also lead to low uptake of screening and immunisation programmes and inadequate support or cultural insensitivity from health care providers (Jayaweera and Quigley 2010; Johnson 2006). A further argument for deteriorating health over time amongst immigrants is that worsening physical health and, in particular, adopting poorer health behaviours, may be compounded by the age of arrival. Specifically, immigrants may be exposed for the first time to unhealthy behaviours, such as smoking, at critical moments in their life course or development when the adoption of such behaviours is more likely. Early age immigration has been found to be associated with higher risk of breast cancer (John et al. 2005) and low offspring birthweight (Bates and Teitler 2008), whilst later age immigration has been associated with poorer self-assessed health (Angel et al. 2001).

The evidence for a healthy immigrant effect in the UK has recently been described as ‘limited’, but nonetheless, it has been reported that ‘most migrants have relatively good health but that the health status of some migrants deteriorates after arrival’ (Jayaweera and Quigley 2010, p. 1003). Recent commentators have noted that families, women and children, are particularly susceptible to poor health due to vulnerability and abuse suffered before and during migration or due to low uptake of health services afterwards (Jayaweera 2014). Evidence for differences between economically better-off and worse-off migrants is said to be particularly lacking (Jayaweera 2014). With regard to recent arrivals, one exception to the healthy migrant thesis is the higher rates of infectious diseases such as HIV and TB amongst migrants (HPS 2011). As for health trajectories after arrival, a study of mothers in the Millennium Cohort Study looked at four health indicators and found some evidence of a healthy immigrant effect; birth abroad was associated with less depression and lower rates of smoking amongst mothers, whilst length of residence in the UK was associated with poorer general health and increasing depression but not with rates of smoking and drinking (Jayaweera and Quigley 2010). With regard to accessing health services, it has been reported that migrant mothers access antenatal care later compared with UK-born mothers (Lewis 2011), although, where no antenatal care was used, socio-demographic factors are stronger predictors than either country of origin or length of residence in the UK (Jayaweera and Quigley 2010). Stronger evidence for the effects of length of residence in the UK has been found for the largest settled ethnic minority group, South Asians. For this group, an epidemiological transition is said to have taken place (Justin et al. 2007), whereby the health advantage of lower rates of cancer than the general population (Harding and Rosato 1999) is being eroded as all-cancer mortality amongst South Asians increases with length of residence in the UK (Harding 2003) and rates of breast cancer for women and lung cancer for men are on the increase in South Asians (Velikova et al. 2004; Smith et al. 2003).

### New Immigration

Migration to the UK has changed in the past few decades in ways which may have implications for immigrant health. So-called ‘new migration’ has been occurring since the 1990s with increased numbers and greater diversity of places of origin, of reasons for migration and of channels of mobility (Finney and Simpson 2009; Koser and Lutz 1998; Vertovec 2007). New migrants to the UK are often seen as comprising two groups, immigrants fleeing conflict in their home countries, and who may have suffered persecution, mostly from African, Middle Eastern and Asian countries, and economic migrants from the Central and East European accession countries after 2004 and 2007, as well as from other parts of the world. Both groups are at risk of poor health, as explained below, although the boundary between them is not watertight since ‘new migrants’ are themselves diverse; for example, European migrants include large numbers of Roma who migrate to escape poverty, discrimination and social exclusion, rather than primarily for employment reasons (SMG 2013).

The first group, asylum seekers and refugees, are said to be disadvantaged ‘because they are forced to migrate and are less able to choose where to live on the basis of availability of social networks’ (Cheung and Phillimore 2013, p. 5). They have often been dispersed to towns and cities with little previous experience of immigration (Jayaweera and Gidley 2011), which may cause them to be exposed to higher levels of harassment and racism and problems of integration. They may be further disadvantaged, or at least face obstacles to social integration, due to having different rights and entitlements to other residents in the areas where they are settled, which either make them standout or cause resentment. These circumstances can affect how other people view them and the respect afforded to them (Ager and Strang 2008). Some time ago, it was recognised that the long period of time taken to decide on asylum cases (described as ‘up to 2 years’ in Sweden, although many recent cases in the UK may have waited a lot longer even than that) was damaging to physical and mental health (Forsberg et al. 1993). Further, the long wait for refugee status, along with the memories of pre-migration traumas, is said to expose refugees to the risk of a later psychiatric crisis, with worse mental health where there are enduring post-migration contextual stressors (Porter and Haslam 2005). Despite the recognition of these issues, the effects of time upon refugee health have rarely been studied, with little if any longitudinal research.

The second group of new migrants—economic migrants coming mainly from Central and Eastern Europe—may comprise lower-wage, skilled manual workers with poor health, in contrast to the self-selecting, high-skilled, high-status immigrants the UK wishes to attract from the rest of the world. Robinson and Reeve (2006) have speculated as to whether new immigrant groups will operate as ‘footloose’ ‘spatial pioneers’, not necessarily tied to existing locations of immigrant settlement, or whether they will form new clusters of settlement, perhaps consisting of diverse nationalities rather than the more homogenous migrant clusters formed in the past. This may affect their ability to access supporting social networks which are often protective of health. Cook et al. (2012) argue that EU A8 migrants are settling in ‘gateway’ neighbourhoods where other similar migrants are located.

Using data from the most recent UK Census, Jivraj and Khan (2013) show that ethnic minorities (not limited to recent migrants but including most migrants) are more likely to live in deprived neighbourhoods than white British people, and it is known that people living in deprived neighbourhoods are more than twice as likely to be unemployed as those living in other neighbourhoods. It is therefore plausible that the health of migrants may be poorer as a result of living in deprived areas. However, there is very little direct evidence on how area of residence impacts on the health of immigrants either in the short or longer term.

## Study Context: Glasgow

Glasgow is a post-industrial city in the west of Scotland, UK. It suffered job losses in the 1980s due to a shakeout of heavy industry and manufacturing, so that today, around one tenth of the city’s jobs are in manufacturing and construction and the largest employment sectors are health services, business administration and retail (Glasgow Marketing Board 2013). The city suffered several decades of population decline before experiencing a small increase in population in recent years, reaching a total population of 593,245 at the 2011 Census, far below its peak of around 1.1 million in the first half of the twentieth century. As well as being noted for decline in the latter part of the last century, Glasgow has relatively high levels of deprivation and poor health. The city contains the largest share of deprived areas of any town or city in Scotland; 42 % of the city’s neighbourhoods were classified as deprived in 2012, a drop from 54 % a decade earlier, but nonetheless a higher rate of deprivation than anywhere else in the country (Scottish Government 2012). There is a 15-year gap in life expectancy between the richest and poorest parts of the city (McCartney 2011). Moreover, Glasgow’s health is worse than expected on the basis of its socio-economic profile, and there have been attempts to explain the city’s excess mortality (Walsh et al. 2010).

Over the last 15 years, Glasgow has also experienced change as a result of new migration. Although the city has had a settled and growing Asian community since the mid-twentieth century (McGarrigle and Kearns 2009), Glasgow’s overall ethnic minority population has traditionally been small for a UK city of its size. In 2001, only 7.2 % of the city’s population (ca. 42,000 people) were classified as ethnic minority (i.e. not white Scottish, British or Irish), but by 2011, this had more than doubled to 15.4 % (ca. 92,000), a rate of increase higher than the national average (Freeke 2013). As a result, more parts of the city have become ethnically diverse. Of the city’s 56 planning neighbourhoods, 5 had an ethnic minority population of 12 % or more in 2001, whilst 11 did so in 2011, including 9 neighbourhoods with an ethnic minority population of between a quarter and a half.

There are three main sources for the increase in migrant population in Glasgow since the millennium. At a national level, the Scottish Government has adopted a pro-immigration strategy, often in contrast to the UK government. In 2004, the Scottish Executive first launched its ‘Fresh Talent’ initiative to attract highly skilled and entrepreneurial individuals to apply for UK work permits after studying at a Scottish university (Scottish Executive 2004). The scheme applied mostly to people outside the EU (Williams and De Lima 2006) and was deemed successful in boosting self-employment and entrepreneurial activity (Houston et al. 2008). The Scottish Government has also taken ‘a symbolically different approach to refugee integration’ by using its powers (in relation to health care, education, housing and language training) ‘to encourage and facilitate integration as soon as an asylum seeker arrives in Scotland’ (Mulvey 2015, p. 365). The official position on immigration in Scotland is often said to reflect public attitudes, but whilst those attitudes can be said to be relatively positive compared to the rest of the UK, it is nonetheless the case that recent studies have shown that a majority of people in Scotland support reduced immigration, with skilled workers and students being the exception (Blinder 2014). Moreover, there is growing opposition to immigration amongst disadvantaged groups such as those with low educational attainment (McCollum et al. 2014).

At the same time, as seeking out global graduates, Glasgow, like other UK cities, has attracted EU migrants after the enlargements of 2004 and 2007. This has led to growth in the Polish and Roma communities in particular, but whilst the former have resided across a number of areas of the city, there has been growth of a large Roma community (said to number 4000) in one neighbourhood which has resulted in ongoing difficulties of integration and discrimination (Poole and Adamson undated; Social Marketing Gateway 2013). The majority of European migrants live in private rented housing, which has also doubled in size over a decade to represent nearly a fifth of the housing market (Scottish Government 2015; Freeke 2012a).

The third component of Glasgow’s increased immigrant population consists of asylum seekers and refugees, reflecting growing global levels of forced displacement (UNHCR 2015). Since 2000, Glasgow City Council has been receiving asylum seekers under contractual arrangements with the UK Home Office within the terms of the Immigration and Asylum Act 1999. Glasgow is the largest ‘dispersal site’ outside London; the first contract from 2000 to 2006 involved 2500 units of social housing and the second contract from 2006 to 2011 was for 5800 bedspaces. It was recorded that 6000 asylum seekers were present in the city in 2003 and 2800 in 2010 with the annual rate of arrival dropping to 1300 in 2008/2009 and again to 700 in 2009/2010 (Freeke 2012b). The number of asylum seekers remaining in the city as refugees after receiving leave to remain is unknown. The majority of asylum seekers are from African and Middle Eastern countries experiencing conflict, with approximately 40 % coming from the following five countries: Iran, Pakistan, Iraq, Somalia and Democratic Republic of Congo (ODS 2007). Ninety percent of asylum seekers were accommodated in just half a dozen locations around the city. The available accommodation has tended to be in low-demand areas, so as not to impact upon the housing waiting list for locals (Crawford et al. 2012). These were mostly high-rise estates of poor quality accommodation in run-down environments. The impacts of asylum seekers and refugees upon receiving communities can be great, particularly where change happens quickly and unexpectedly; in the early years of the resettlement programme, there were a high number of incidents of racial harassment on some of the estates (Binns 2002), indicating that community cohesion was an issue. As a result, integration networks were established in key parts of the city to support migrants and aid the development of cohesion. Funding for integration activities totalled nearly £1 million per annum until 2010/2011 when funding was severely cut (Scottish Government 2003; ODS Consulting 2007).

## Research Aims

Our aim was to investigate the health of migrants living in deprived areas of a post-industrial, regional city with particular emphasis on the effects of time. More specifically, we sought to address the following questions:

1. How does migrant health compare to that of the British-born or indigenous population; specifically, are migrants healthier, in line with the healthy migrant thesis?
2. What is the effect on migrant health of time spent in the UK? In particular, is there evidence of a decline in health over time, in line with the healthy migrant thesis, and are the effects of time different for social and economic migrants than for asylum seekers and refugees?
3. For refugees, are the effects on health different between the duration of time before and after the leave-to-remain decision?
4. What is the impact on migrant health of the time spent living in a (deprived) area, and is this the same or different for migrants as for British-born?

## Methods

### Data Source

The data come from a study of the effects of housing investment and regeneration on health and well-being in Glasgow (Egan et al. 2010). The 15 study communities all fall within the 15 % most deprived in Scotland, often targeted for area-based initiatives to tackle disadvantage (Walsh 2008). Surveys conducted in the 15 communities in 2008 and 2011 have been combined to form the data set for this analysis. A random sample of addresses were selected in nine of the communities and in six communities (subject to regeneration/redevelopment); all existing addresses were included in the survey. One adult respondent was interviewed at each address. The response rates to the surveys were 47.5 % in 2008 and 45.4 % in 2011. All respondents were asked to describe their current legal status as residents. From this, we were able to identify two migrant samples. First, asylum seekers who had applied for, and refugees, who had received, leave to remain in the UK.^1^ This group were mostly from Africa and Asia. Second, other migrants including those born abroad who now held British citizenship, economic migrants and foreign students. We call this group ‘social and economic migrants’, also containing significant numbers from African and Asia, but in addition a large number of white people, the largest group coming from Europe. As a comparison for the effects of time in the area, we use the remainder of the sample, the British-born. People who participated in more than one survey were included only once; sensitivity analysis confirmed that the choice of survey did not affect the results, and so, a random selection of survey was made.

### Health Indicators (Dependent Variables)

We examined four health outcomes. Self-rated health was based on respondents’ responses to “In general, would you say your health is excellent, very good, good, fair, poor”, coded as ‘good’ or better versus fair or poor. Physical and mental health were assessed using the SF-12v2 Physical and Mental Health Component Scores (SF-12 PCS and SF-12 MCS). The SF-12v2 is a validated questionnaire for measuring health-related quality of life; scores are computed from responses to 12 questions and range from 0 to 100, with higher scores indicating better health (Ware et al. 2005). The SF-12v2 is based on the longer SF-36, which has been translated and used in over 40 countries across several continents, with validation for use in multinational clinical trials across 14 countries (Ware and Gandek 1998). Finally, mental well-being (positive affect and positive function) was assessed using the Warwick-Edinburgh Mental Well-Being Scale (WEMWBS), which is based on responses to 14 questions to produce a score from 14 to 70, with higher values indicating better mental well-being (Tennant et al. 2007). WEMWBS has been validated for cross-cultural use in the UK, with Chinese and Pakistani ethnic groups (Taggart et al. 2013). Neither health scale has been validated specifically for use with the range of ethnic/migrant groups in this study, although they have been used with a wide range of ethnicities.

### Time Measures (Potential Explanatory Variables)

Four time variables were used in the analysis. First, all migrant respondents were asked for the month and year of their arrival in the UK, from which we calculated their duration of stay in the UK in years up to the date of the interview. We divide this duration into the following four categories for use in the analysis: 2 years, 2–5 years, 6–10 years and 11+ years. Second, all respondents were asked how long they had lived in their current area in year bands, which we collapsed into the following four categories: <1 year, 1–2 years, 3–5 years and 6+ years. Finally, those asylum seekers who had received leave to remain were asked the month and year of that decision, from which we calculated their time in the UK before and after achieving refugee status.

### Socio-Demographic Controls

As health outcomes might be expected to worsen with advancing age, we adjusted analyses for age at the time of interview (age groups 16–24, 25–39, 40+). We also adjusted for gender, household type (adult only, family with dependent children, older person(s)), education (none/school leaving certificate (SLC)^2^, higher than SLC), employment (working, not working, retired), and difficulties speaking English (assessed by interviewer yes, no). The last of these is used as it may influence migrants’ access to health services, something which has been remarked on previously (Mladovsky 2007).

### Analysis

We begin by comparing the four health outcomes across the three study groups (British-born, plus two migrant groups) by household type, reflecting earlier findings on families (Jayaweera 2014). Here, we compare the proportions reporting good or better health and the mean scores on the other three outcomes.

We conduct logistic (self-rated health) and least squares (physical/mental health, well-being) regression analyses for the effects of time in the UK upon each of the health outcomes separately for social and economic migrants and for asylum seekers and refugees. We present odds ratios for better self-rated health and differences in mean scores for the following three scales: SF-12 PCS, SF-12 MCS and WEMWBS. Ninety-five-percent confidence intervals are also presented along with *p* values for linear trend across the time categories, as we might expect linearity in the effects of time, based on the existing literature. Similar analyses are then conducted for the refugee group only, using time without and time after leave to remain as independent variables in two sets of models. Finally, we conduct regression analysis on the four health outcomes again, using time living in the area as the independent variable. This analysis is performed separately for the three study groups—UK-born, social and economic migrants and asylum seekers and refugees—and results compared. We present unadjusted analysis first and then analysis adjusted for all the socio-demographic controls. Unadjusted analyses were based on respondents with complete data for all covariates of interest to allow comparison of results. In interim analyses, we also adjusted for each control in turn, to look at the individual effects, and whilst we comment on this in the results, for simplicity, we do not explicitly present individual adjustments.

## Results

### Characteristics of Migrants Compared with British-Born

The number of respondents varies between the outcomes examined, but as shown in Table 1, the maximum numbers used in the analysis are 5787 British-born, 712 social and economic migrants and 692 asylum seekers and refugees (including 336 refugees). Migrants tend to be younger than the British-born residents, with twice as many respondents in the young adult age group (16–24). More of the social and economic migrants are middle-aged or older (aged 40+) than asylum seekers and refugees, though still far fewer than amongst the British-born. The two migrant samples contain two to three times as many families with dependent children, compared with the British-born households, reflecting the differences in age. Social and economic migrants were more likely to have post-school educational qualifications than either British-born or asylum seeker and refugee respondents. Social and economic migrants were also the most likely to be working and asylum seekers and refugees the least likely; a third of the British-born were retired, some of whom were at pre-retirement age. Two thirds of each of the migrant groups were deemed to have no difficulty speaking English.

**Table 1:** Characteristics of native and migrant groups

	UK-born (*n* = 5787)	Social and economic migrants (*n* = 712)	Asylum seekers and refugees (*n* = 692)
Gender *n* (%)			
Male	2424 (41.9)	332 (46.6)	277 (40.0)
Female	3363 (58.1)	380 (53.4)	415 (60.0)
Age group *n* (%)			
16–24	375 (6.5)	100 (14.0)	109 (15.8)
25–39	1254 (21.7)	366 (51.4)	405 (58.5)
40+	4158 (71.9)	246 (34.6)	178 (25.7)
Household type *n* (%)			
Adult	2671 (46.2)	308 (43.3)	255 (36.9)
Family (with children)	1385 (23.9)	373 (52.4)	430 (62.1)
Older	1731 (29.9)	31 (4.4)	7 (1.0)
Education *n* (%)			
None/SLC	3839 (66.3)	358 (50.3)	483 (69.8)
Higher than SLC	1948 (33.7)	354 (49.7)	209 (30.2)
Employment *n* (%)			
Working	1482 (25.6)	282 (39.6)	110 (15.9)
Not working	2449 (42.3)	397 (55.8)	573 (82.8)
Retired	1856 (32.1)	33 (4.6)	9 (1.3)
Difficulties with English *n* (%)			
No	5394 (93.2)	484 (68.0)	452 (65.3)
Yes	393 (6.8)	228 (32.0)	240 (34.7)

Based on those cases with complete data for self-rated health

Table 2 compares the four health outcome measures between the three study groups, by household type. In almost every case, the health indicator is worse for the British-born respondents compared with the two migrant groups, suggesting that migrants are healthier than indigenous residents within each household type. This finding reflects not only the health status of more recent migrants but also the health of migrants who had been in the UK for longer, since nearly three fifths of social and economic migrants and two fifths of asylum seekers and refugees had been in the UK for over 5 years. The gap between migrants and British-born is particularly large for self-rated health; e.g. a quarter more migrants than British-born adult households rate their health as good or better. Across all four outcomes, younger respondents (i.e. not ‘older households’) from the two migrant groups are similar to each other and healthier than indigenous respondents, particularly so in the case of households without children (‘adult households’). For older households, social and economic migrants are more similar to the British-born, and older asylum seekers and refugees are the healthiest group.

**Table 2:** Health status of native and migrant groups, by household type

	UK-born	Social and economic migrants	Asylum seekers and refugees	*P* value*
Self-rated health: proportion of respondents with good, very good, or excellent health (95 % CI)				
Adult household	0.60 (0.58, 0.62)	0.86 (0.83, 0.90)	0.85 (0.81, 0.90)	<0.001
Family household	0.77 (0.75, 0.79)	0.87 (0.83, 0.90)	0.88 (0.85, 0.91)	
Older household	0.52 (0.49, 0.54)	0.58 (0.41 (0.75)	0.86 (0.60, 1.12)	
Physical health: SF-12 PCS mean score (95 % CI)				
Adult household	45.1 (44.6, 45.5)	52.0 (50.6, 53.4)	52.8 (51.2, 54.3)	<0.001
Family household	50.8 (50.1, 51.4)	51.7 (50.4, 52.9)	52.7 (51.5, 53.9)	
Older household	38.9 (38.3, 39.5)	38.0 (33.5, 42.6)	47.3 (38.1, 56.6)	
Mental health: SF-12 MCS mean score (95 % CI)				
Adult household	46.9 (46.5, 47.3)	51.2 (50.0, 52.5)	50.7 (49.4, 52.1)	<0.001
Family household	47.9 (47.4, 48.5)	50.4 (49.3, 51.6)	50.2 (49.1, 51.2)	
Older household	51.1 (50.6, 51.7)	51.3 (47.3, 55.4)	56.0 (47.7, 64.3)	
Mental well-being: WEMWBS mean score (95 % CI)				
Adult household	49.6 (49.2, 50.0)	54.0 (52.8, 55.1)	52.5 (51.3, 53.8)	<0.001
Family household	51.8 (51.3, 52.3)	53.3 (52.2, 54.3)	51.2 (50.2, 52.2)	
Older household	50.2 (49.8, 50.7)	50.9 (47.3, 54.5)	56.0 (48.4, 63.6)	

*p for interaction

### The Effects of Time in the UK on Migrant Health and Well-Being

Tables 3 and 4 give the results of the regression models for the effects of time in the UK on the four health indicators for social and economic migrants and asylum seekers and refugees, respectively. Nearly three in five social and economic migrants have lived in the UK for over 5 years and a fifth for over a decade (Table 3). Results demonstrate a consistent worsening of health with increasing time spent in the UK (e.g. OR (95 % CI) for good or better self-rated health 0.55 (0.15, 1.94), 0.21 (0.06, 0.69) and 0.10 (0.03, 0.32), respectively, for respondents resident in the UK for 2–5, 6–10 and 11+ versus <2 years). For all four health indicators, there was evidence of a ‘dose-response’ association, with progressively poorer health outcomes in respondents who had spent longer in the UK. Adjustment for socio-demographic characteristics, particularly age and employment status, somewhat attenuated associations, most markedly those with physical health and mental well-being. However, even after adjustment for all socio-demographic characteristics, strong associations of worsening health with increasing time in the UK remained.

**Table 3:** Effects of time in UK on health indicators for social and economic migrants

	Number (%)	Unadjusted	Adjusted^a^
Odds ratio for better self-rated health (95 % CI)			
<2 years	78 (11.0)	1.00	1.00
2–5 years	229 (32.2)	0.55 (0.15, 1.94)	0.58 (0.16, 2.18)
6–10 years	267 (37.6)	0.21 (0.06, 0.69)	0.27 (0.08, 0.96)
11+ years	137 (19.3)	0.10 (0.03, 0.32)	0.18 (0.05, 0.67)
*P (trend)* ^b^		*<0.001*	*<0.001*
Difference in SF-12 PCS score (95 % CI)			
<2 years	78 (11.0)	0.00	0.00
2–5 years	229 (32.2)	−1.33 (−3.88, 1.21)	−0.53 (−2.90, 1.84)
6–10 years	267 (37.6)	−3.98 (−6.48, −1.48)	−2.03 (−4.40, 0.35)
11+ years	137 (19.3)	−9.07 (−11.80, −6.31)	−4.21 (−6.92, −1.50)
*P (trend)* ^b^		*<0.001*	*<0.001*
Difference in SF-12 MCS score (95 % CI)			
<2 years	78 (11.0)	0.00	0.00
2–5 years	229 (32.2)	−1.71 (−4.31, 0.88)	−1.50 (−4.15, 1.16)
6–10 years	267 (37.6)	−3.76 (−6.30, −1.21)	−2.95 (−5.61, −0.29)
11+ years	137 (19.3)	−4.73 (−7.44, −1.82)	−3.69 (−6.72, −0.65)
*P (trend)* ^b^		*<0.001*	*<0.001*
Difference in WEMWBS score (95 % CI)			
<2 years	78 (11.0)	0.00	0.00
2–5 years	229 (32.2)	−2.44(−4.93, 0.05)	−2.20 (−4.72, 0.32)
6–10 years	267 (37.6)	−4.27 (−6.72, −1.83)	−3.30 (−5.81, −0.78)
11+ years	137 (19.3)	−6.35 (−9.04, −3.65)	−4.55 (−7.43, −1.67)
*P (trend)* ^b^		*<0.001*	*<0.001*

^a^Adjusted for gender, age group, household type, education, employment, and difficulties with English

^b^ *P* values are for incremental change in odds ratios for health indicators across time categories

**Table 4:** Effects of time in UK on health indicators for asylum seekers and refugees

	Number (%)	Unadjusted	Adjusted^a^
Odds ratio for better self-rated health (95 % CI)			
<2 years	103 (14.5)	1.00	1.00
2–5 years	337 (47.5)	1.54 (0.80, 2.96)	1.63 (0.80, 3.34)
6–10 years	239 (33.7)	1.11 (0.57, 2.16)	1.39 (0.66, 2.89)
11+ years	31 (4.4)	0.68 (0.24, 1.96)	0.86 (0.26, 2.80)
*P (trend)* ^b^		*0.47*	*0.98*
Difference in SF-12 PCS score (95 % CI)			
<2 years	103 (14.5)	0.00	0.00
2–5 years	337 (47.5)	0.73 (−1.46, 2.93)	0.49 (−1.67, 2.65)
6–10 years	239 (33.7)	−0.57 (−2.85, 1.72)	−0.36 (−2.65, 1.93)
11+ years	31 (4.4)	−0.23 (−4.21, 3.78)	0.37 (−3.56, 4.29)
*P (trend)* ^b^		*0.37*	*0.67*
Difference in SF-12 MCS score (95 % CI)			
<2 years	103 (14.5)	0.00	0.00
2–5 years	337 (47.5)	−1.10 (−3.45, 1.25)	−0.67 (−3.04, 1.69)
6–10 years	239 (33.7)	−0.35 (−2.79, 2.09)	0.44 (−2.07, 2.94)
11+ years	31 (4.4)	−2.33 (−6.59, 1.93)	−1.45 (−5.74, 2.84)
*P (trend)* ^b^		*0.71*	*0.79*
Difference in WEMWBS score (95 % CI)			
<2 years	103 (14.5)	0.00	0.00
2–5 years	337 (47.5)	−0.75 (−3.00, 1.50)	−0.46 (−2.64, 1.72)
6–10 years	239 (33.7)	−0.43 (−2.78, 1.91)	0.15 (−2.17, 2.47)
11+ years	31 (4.4)	−0.89 (−4.99, 3.21)	−1.88 (−5.87, 2.10)
*P (trend)* ^b^		*0.80*	*0.87*

^a^Adjusted for gender, age group, household type, education, employment, and difficulties with English

^b^ *P* values are for incremental change in odds ratios for health indicators across time categories

Asylum seekers and refugees have generally been in the UK for less time than social and economic migrants, with two in five residing for more than 5 years and only 4 % being in the UK for over a decade (Table 4). In contrast to results for social and economic migrants, there was no strong evidence of any associations of time in the UK with health indicators for asylum seekers and refugees. Effect sizes were generally small or inconsistent, confidence intervals all comfortably include 1 or 0 (no association) and *p* values for trend across categories of time were large.

### The Effects of Time Before and After Leave to Remain on Refugee Health and Well-Being

Two thirds of refugees had waited 1–4 years for a leave-to-remain decision, with a third waiting 5 or more years (Table 5). Following that decision, only a fifth of refugees have lived in the UK for a further 5 years, with the majority residing for less time than that. Results from regression analyses are characterised by relatively wide confidence intervals as a result of the smaller sample of refugees compared with the larger sample of asylum seekers and refugees combined and associations do not generally reach conventional levels of statistical significance. However, in the context of these relatively small numbers, both health and well-being appear to decline, at least to some extent, with time spent waiting for a decision on an asylum application, particularly when waiting for 5 or more years. In contrast, the results suggest that, if anything, self-rated health, physical health and mental health improve slightly with time after leave to remain, particularly in the first 4 years. Mental well-being continues to decline with time after leave to remain, although less markedly than with time waiting for leave to remain.

**Table 5:** The effects of time before and after leave to remain on health indicators for refugees

	Time without leave to remain	Time since leave to remain
	*n* (%)	
<1 year	106 (31.8)	113 (33.6)
1–4 years	119 (35.7)	151 (44.9)
5+ years	108 (32.4)	72 (21.4)
Odds ratio for better self-rated health (95 % CI)		
<1 year	1.00	1.00
1–4 years	0.70 (0.26, 1.87)	1.46 (0.65, 3.29)
5+ years	0.45 (0.18, 1.16)	1.01 (0.41, 2.49)
*P (trend)* ^a^	*0.09*	*0.86*
Difference in SF-12 PCS score (95 % CI)		
<1 year	0.00	0.00
1–4 years	−0.71 (−3.23, 1.81)	1.05 (−1.27, 3.38)
5+ years	−2.54 (−5.15, 0.06)	0.07 (−2.93, 2.79)
*P (trend)* ^a^	*0.05*	*0.09*
Difference in SF-12 MCS score (95 % CI)		
<1 year	0.00	0.00
1–4 years	0.02 (−2.76, 2.79)	0.66 (−1.89, 3.22)
5+ years	−0.65 (−3.52, 2.22)	−0.01 (−3.16, 3.13)
*P (trend)* ^a^	*0.65*	*0.93*
Difference in WEMWBS score (95 % CI)		
<1 year	0.00	0.00
1–4 years	−1.40 (−3.94, 1.15)	−0.65 (−3.00, 1.70)
5+ years	−1.68 (−4.31, 0.94)	−1.01 (−3.91, 1.89)
*P (trend)* ^a^	*0.21*	*0.47*

All results adjusted for gender, age group, household type, education, employment, and difficulties with English

^a^ *P* values are for incremental change in odds ratios for health indicators across time categories

### The Effects of Time Spent Living in a Deprived Area on Resident Health and Well-Being

The vast majority (three quarters) of UK-born respondents had lived in their area of residence for more than 5 years, compared with just over a quarter of social and economic migrants, and one in six asylum seekers and refugees, with half the latter group living in their area for only 1 or 2 years (Table 6). For the UK-born, self-rated health, well-being and particularly, physical health are worse for those living in the area more than 1 year, compared with those recently arrived in the area, although there is little, if any, evidence for any further decline thereafter. In contrast, there is, if anything, a slight increase in mental health score in UK-born respondents who lived in the area for 6+ years, although this is marginal. For social and economic migrants, both self-rated health and physical health decline somewhat with time beyond 2 years spent living in the area, although this pattern is not consistent across all time categories. There is no association with time in the area for mental health or mental well-being for this group. In the case of asylum seekers and refugees, those who have lived in the area for more than a year have somewhat better self-rated health and better mental well-being than recent arrivals, although the confidence intervals are wide and there is no clear pattern of subsequent improvement with time. There is no marked association with time in the area for physical or mental health for the asylum seeker and refugee group.

**Table 6:** The effects of time spent living in the area on health indicators for native and migrant groups

	UK-born	Social and economic migrants	Asylum seekers and refugees
Time in area		*n* (%)	
<1 year	304 (5.0)	115 (15.4)	165 (22.8)
1–2 years	487 (8.0)	173 (23.2)	197 (27.2)
3–5 years	664 (10.8)	249 (33.3)	240 (33.1)
6+ years	4,668 (76.2)	210 (28.1)	123 (17.0)
Odds ratio for better self-rated health (95 % CI)			
<1 year	1.00	1.00	1.00
1–2 years	0.66 (0.45, 0.95)	1.13 (0.44, 2.88)	1.61 (0.80, 3.25)
3–5 years	0.70 (0.50, 0.98)	0.51 (0.23, 1.13)	1.31 (0.69, 2.49)
6+ years	0.73 (0.54, 0.98)	0.42 (0.19, 0.94)	1.59 (0.73, 3.45)
*P (trend)* ^a^	*0.32*	*0.01*	*0.35*
Difference in SF-12 PCS score (95 % CI)			
<1 year	0.00	0.00	0.00
1–2 years	−2.09 (−3.97, −0.21)	0.79 (−1.41, 2.99)	0.31 (−1.68, 2.30)
3–5 years	−2.87 (−4.60, −1.15)	−0.95 (−3.02, 1.12)	−0.84 (−2.76, 1.09)
6+ years	−2.96 (−4.48, −1.45)	−1.59 (−3.82, 0.64)	−0.51 (−2.82, 1.79)
*P (trend)* ^a^	*<0.001*	*0.04*	*0.38*
Difference in SF-12 MCS score (95 % CI)			
<1 year	0.00	0.00	0.00
1–2 years	−0.18 (−1.90, 1.53)	0.40 (−2.03, 3.82)	0.75 (−1.44, 2.93)
3–5 years	0.55 (−1.02, 2.13)	−0.32 (−2.60, 1.96)	−1.05 (−3.16, 1.05)
6+ years	1.00 (−0.38, 2.38)	0.42 (−2.04, 2.87)	−1.28 (−3.81, 1.25)
*P (trend)* ^a^	*0.03*	*0.93*	*0.13*
Difference in WEMWBS score (95 % CI)			
<1 year	0.00	0.00	0.00
1–2 years	−2.28 (−3.80, −0.76)	0.25 (−2.05, 2.55)	0.24 (−1.82, 2.29)
3–5 years	−1.50 (−2.89, −0.10)	−0.84 (−3.01, 1.34)	1.31 (−0.67, 3.28)
6+ years	−0.88 (−2.10, 0.34)	0.20 (−2.14, 2.54)	1.07 (−1.31, 3.46)
*P (trend)* ^a^	*0.55*	*0.86*	*0.20*

All results adjusted for gender, age group, household type, education, employment, and difficulties with English

^a^ *P* values are for incremental change in odds ratios for health indicators across time categories

## Discussion

Returning to the aims of the research and the first component of the healthy migrant thesis, we found that migrants in Glasgow tend to be healthier than their indigenous co-residents across four health outcome measures. Moreover, this is not simply a question of the confounding effects of younger ages; whilst migrants in adult households below retirement age are healthier than the UK-born, so too are those from older person households in the asylum seeker/refugee group, echoing an earlier finding for older migrants in Canada (Gee et al. 2003). The fact that this healthy migrant effect is seen in asylum seekers and refugees as well as amongst social and economic migrants suggests that the effect is not solely a consequence of self-selection on the part of migrants, nor of screening processes instituted by the receiving country, since under Home Office arrangements, Scotland does not select those asylum seekers arriving north of the border. Indeed, in contrast to previous explanations for the healthy migrant effect which focus on characteristics of the country of origin (e.g. Dunn and Dyck 2000), our results also highlight the importance of the country of destination; if migrants arrive or settle in an unhealthy city, and, moreover, in the more deprived parts of that city, then a healthy migrant effect may become apparent as a consequence. This is somewhat different to the situation that may be found in other, healthier destinations. For example, the decline over time in health satisfaction amongst East European migrants to Germany has been posited to result from ‘the unfavourable health conditions and high prevalence of risk factors that immigrants were exposed to in Eastern Europe’, i.e. their country of origin (Ronellenfitsch and Razum 2004, p. 5).

However, although migrants appear to offer a potentially beneficial human capital resource to deprived areas, our findings also suggest that access to education, English language tuition and employment opportunities are issues for both of the migrant groups and likely barriers to their positive contribution to society and the economy (see also Kearns and Whitley 2015). This may also be of broader concern, given recent findings from Sweden that those migrants with weak labour market attachment (measured in terms of education, income and employment) have higher rates of severe morbidity than the indigenous population (Klinthall 2008).

With regard to the second element of the healthy migrant thesis, and our second research aim, we found that the health of social and economic migrants deteriorates with time spent in the UK, but this was not the case for asylum seekers and refugees, where no clear pattern was found. This is an important distinction, reflecting earlier comments that the effects of migration upon health will vary between migrant groups, partly due to the different migration processes experienced (Rechel et al. 2013). Such a clear difference between the two migrant groups in our study is indicative of the negative effects being produced by social contact with the indigenous population, as social and economic migrants comprise to a large extent people who are working or studying which brings them into contact with British-born residents, whereas these activities are restricted and far less common amongst asylum seekers and refugees. A survey of refugees in 2010 found that very few people were in employment (<15 %) and the vast majority (85 %) wanted to return to education (Mulvey 2011), although waiting lists, childcare needs and costs were barriers (Mulvey 2013). Previous analysis has shown that several indicators of social relations are lower for asylum seekers and refugees than for other migrants, including in particular indicators of interactions with neighbours in the local area and use of local social amenities (Kearns and Whitley 2015). However, social interaction and acculturation are not the only potential explanations for worsening migrant health amongst some groups; structural factors such as poor housing and neighbourhood quality can have direct effects upon migrant health as well as being the ‘means’ to social connections and cultural ‘facilitators’ of integration (Ager and Strang 2004). In our current sample, the rate of employment is twice as high amongst social and economic migrants as amongst asylum seekers and refugees; moreover, Census results for Scotland show that over 70 % of European migrants (unlikely to be refugees) are either employed or studying (Scottish Government 2015). The question of whether such functional factors or ‘markers of integration’ (Ager and Strang 2004) can have negative effects upon migrant health over time is not often considered.

We cannot tell whether the decline in health over time for social and economic migrants is the result of acculturation and the adoption of indigenous lifestyles and health behaviours (Berry 1997), but this is plausible, since such lifestyle factors are recognised as being relatively poor in Scotland by international or European standards (Leon et al. 2003). However, although linear acculturation models have been questioned (Jayaweera 2014), our results showed consistent trends of declining health with increasing time in the UK for all four health indicators, which supports the notion of a ‘strong temporal component to migrant health’. Although we have not been able to investigate any separate life course effects alongside the effects of time (Rechel et al. 2013, p. 1239), ours is the first study for over 20 years to examine the effects of length of residence upon the health of migrants in Glasgow and uses a more refined measure of time than the earlier study of the settled South Asian population in the city (Williams 1993). Our results are consistent with this earlier study, which reported a worsening of health according to time spent in the UK, at least for a quarter of the indicators examined, something we have confirmed and updated for a broader range of migrants.

For our third research question, we investigated the effects of time upon the health of refugees, contrasting the periods before and after receiving a leave-to-remain decision, which we believe to be unique in UK studies. We identified a contrast between deteriorating health with time spent before a leave-to-remain decision and, if anything, improvement with time thereafter, particularly in respect of self-rated health and physical health. We also found that well-being declined for refugees according to time awaiting a decision. These findings are consistent with those of Mulvey (2015), who found asylum seekers’ self-rated health and mental well-being to be lower than that of refugees in Scotland. In addition to adding physical health to this assessment, our findings are based on a sample three times larger than that employed by Mulvey and extend previous results by exploring the effects of duration of status as well as the contrast in status between asylum seekers and refugees. Our findings also suggest that the negative effects of time awaiting a leave-to-remain decision on well-being continue even after the individuals have acquired refugee status, which offers a new understanding of the impacts.

Two explanations have been offered for the negative effects of asylum seeker status upon applicants’ health. First, restrictions on the engagement of asylum seekers with mainstream society, especially through employment and associated socialising (Mulvey 2015; Bloch 2000), has negative effects which are said to be ‘more obvious…for those in the asylum system for longer periods of time’. Second, the situation is said to be further compounded by the reliving of traumatic experiences within a ‘culture of disbelief’ within the asylum process (Souter 2011), which is said to impact upon people’s ability and willingness to be ‘open to new people and experiences’ (Mulvey 2015, p. 13). We have been able to isolate and quantify these process-duration effects and show that their impact is greater after 5 or more years awaiting a decision.

Thereafter, we found that refugees’ well-being may continue to decline after refugee status is obtained. This could be due to what is termed the ‘cessation clause’ introduced in the UK by legislation in 2006 so that asylum seekers are granted temporary leave to remain for 5 years with information on the person’s country of origin reviewed during this time. The consequences of introducing such uncertainty are claimed to be common and described as ‘fear amongst refugees about what will happen if rules change, what will happen at the end of the 5 years and what happens, or pointedly what does not happen, during those 5 years’ (Stewart and Mulvey 2014, p. 1030). Our finding comes from a study which post-dates this change in the rules and, although requiring corroboration in a larger study, is consistent with the notion that uncertainty or fear *after the initial leave-to-remain decision* has a negative impact upon mental well-being. The finding also extends Goldsmith’s view that although refugees may ‘obtain physical security by coming to the UK, they may not immediately experience psychological security’ (Goldsmith 2008, p. 1034).

Our findings on the effects of time spent living in a deprived area are very interesting and have implications for how we understand the role of housing choice, the effects of migrant concentration and the effectiveness of refugee support services. The UK-born are the most negatively affected in health terms by living in a deprived area for more than 1 year; social and economic migrants are also negatively affected but to a lesser extent than the UK-born. These negative effects are seen for both groups in the case of self-rated health and physical health and in addition for mental well-being in the case of the British-born. However, the negative effects on social and economic migrants of living in a deprived area for more than five years are less than the effects of the equivalent time spent in the UK. What is interesting is that these two groups have some degree of choice over where they live, although their choice of residential location is probably constrained, either in the social or private sectors, given that the majority of both groups are not working and thus have limited resources within the housing market. The decline in the health and well-being of the British-born and social and economic migrants may be partly due to environmental conditions and cultural circumstances in deprived areas, as well as being impacted by the negative reputations of some of the areas (Kearns et al. 2013), i.e. by the opportunity structures afforded by the areas’ infrastructural resources, as well as by the areas’ collective social functioning (Macintyre et al. 2002). The constrained choice for migrants, alongside their heterogenous nature in terms of countries of origin, suggests that contextual factors could be an important influence upon their health over time. Other quasi-experimental research with migrants from the Former Soviet Union to Germany has also found that when compositional factors are accounted for, and self-selection of individuals to particular destination locations is ruled out, then contextual factors such as regional incomes, employment and infrastructure are influential upon migrant mortality (Reiss et al. 2013).

In the case of asylum seekers and refugees, the effect of living in the same area beyond 1 or 2 years is positive in the case of two of the indicators, namely self-rated health and mental well-being (albeit with wide confidence intervals). This is despite the fact that this group of migrants live in the most disadvantaged of the study areas, being places of low demand scheduled for wholesale or partial demolition (Crawford et al. 2012). Two possible reasons for these findings are discussed here. First, asylum seekers and refugees have no choice over where they are living, having been placed in their areas of residence under a Home Office settlement programme (Home Office 2000), but at least there are some requirements as to housing standards under such a programme. This suggests the intriguing possibility that a constrained choice of location, as exercised by social and economic migrants, might have more negative effects on health and well-being than having no choice. Social and economic migrants are more likely to end up in poor quality, rented housing where standards are weakly enforced, so-called ‘badly managed lettings, used by migrants’ (Perry 2012, p. 1). Further, part of the negative effect for social and economic migrants may be upon self-perception and well-being, through the realisation that one could only achieve residence in a relatively undesirable place, particularly given the importance of place of residence for psychosocial outcomes (Kearns et al. 2012). This is an alternative paradox of choice to that normally discussed in consumer psychology, whereby too much choice is said to be bad for well-being (Schwartz 2004); i.e. in the case of residential location, no choice might be better than very limited choice for migrants.

The second potential explanation for the findings, again a function of the no-choice location process for asylum seekers and refugees which concentrates the group in a small number of areas, is that strong social networks which have developed amongst this migrant group, plus spatially targeted support programmes, have had a positive and/or protective effect upon the health and well-being of asylum seekers and refugees. This is similar to the social network mechanism suggested in studies of the ‘buffering effects’ of ethnic density against the impacts of racism upon health for ethnic minorities in the UK (Becares et al. 2009). Other recent studies in Scotland have reported ‘generally high levels of social connectedness’ amongst refugees (Strang et al. 2015, p. 46) and ‘a growing sense of pride and attachment to their neighbourhoods’ amongst asylum seekers and refugees in contrast to weak attachment amongst the British-born in the same areas (Egan et al. 2015, p. 105), although this social advantage may be being lost as the estates in question are partially or wholly cleared and residents dispersed . Glasgow also has a number of Refugee Community Organisations and nine Integration Networks, the latter set up over the past 15 years to support asylum seekers and refugees in particular parts of the city, including four which cover the locations in our study where the group live. A recent audit of these networks found that 40 % of their activities were concerned with community activities and social bonds both amongst migrants and between migrants and indigenous residents (CJM 2015).

Both these mechanisms—developing social networks and effective integration support services—may be facilitated by spatial concentration, with four fifths of asylum seekers in the city living in ten postcode districts and half the refugees living in six postcode districts (CJM 2015). The fact that self-rated health and mental well-being appear to improve for asylum seekers and refugees who have lived in their (deprived) areas of residence for more than a year or two, in contrast to opposite effects for British-born and social and economic migrants, suggests that this concentration may be directly and indirectly beneficial for the well-being of this group. This would be in accord with the positive view of migrant concentrations as indicative of strength of community formation (Schrover and Van Lottum 2007), as well as with recent findings from Canada which show that immigrant concentrations do ‘[not] always mean a lack of integration’ and ‘In contrast to many European countries… [are not] seen…as problematic’ (Murdie and Ghosh 2010, p. 308); for asylum seekers and refugees in Glasgow, we would say that the same is true.

### Strengths and Limitations

In common with other studies of this type (Perez 2002), ours contains modest-sized samples of the migrant groups, as a result of which we cannot distinguish migrants’ ethnicity or country of origin for analysis. Religion may also be relevant for some health behaviours that may impact upon other health outcomes, but our survey did not ask about the respondent’s religion. In addition, as our study is based on an interview survey, it has the weakness of relying upon self-reported health indicators. This would have most effect upon the findings if there were an initial under-reporting of health problems by migrants followed by a growing willingness to report health issues over time. We do not know if this is the case, although the legal requirement of asylum seekers and refugees to register with a doctor and the widely known free access to NHS services by both European and non-European economic migrants would suggest that barriers in access to health services are not as great a problem as has been considered elsewhere (McDonald and Kennedy (2004). Finally, we have not been able to test any effects of age of arrival upon the health outcomes examined, only the effects of duration.

Although our study is small in size compared to national-level studies of migrant health, we believe that it has many characteristics that make it distinctive and valuable as a contribution to the evidence on the healthy immigrant effect in the case of the UK, where evidence is sparse. First, it was undertaken in Scotland which has a more pro-immigration stance compared with the rest of the UK. Second, we are able to distinguish between two main classes of migrant, those who come for social and/or economic reasons and those who arrive as asylum seekers. This enables us to examine important differences between migrants who exercise more choice and self-selection about movement (an important factor according to the literature) and migrants who are forced to move due to conditions in their home country. For the latter group, and refugees in particular, we are also able to examine the effects of time spent before and after a leave-to-remain decision. The effects of waiting for such a decision are often remarked upon but the role of the duration of the wait has not previously been examined, and our data provide a unique insight in this regard. Lastly, our examination of the effects of time spent living in a deprived area upon migrant health is novel, and we are also able to compare migrants with UK-born residents living in the same area. Given that living in a deprived area is expected to have detrimental effects, and that migrants often reside in such places, it is important to compare migrants with others in similar residential circumstances; many national studies do not do this.

## Conclusion

We investigated the healthy immigrant effect in Glasgow, Scotland. Whilst we concur with the argument that the country of origin can provide insights into the health effects of migration (Rechel et al. 2013; Spallek et al. 2011), our study has highlighted the effects of the destination upon health outcomes over time for migrants. Glasgow is a city with high levels of deprivation and poor health by Western standards. Arriving migrants are therefore often healthier than the indigenous population, almost irrespective of where they come from. This opens up a new line of inquiry, related, though somewhat different, to the health transition discussed by Razum and Twardella (2002), who argued that a mortality advantage could exist for migrants moving from poorer, less urbanised origin countries to richer, industrialised destination countries since they experience a rapid increase in health service availability, but a slower increase in risks such as for heart disease. In the case of migrants to Glasgow, it may be that the rise in risks from factors such as poor health behaviours is more rapid. Indeed, Spallek et al. (2011) have proposed the development of a better theoretical framework for examining migrant health that considers risks and exposures in both the origin and destination countries, although they also argue for the inclusion of life course and inter-generational factors which we have not considered here. Nonetheless, over time, migrant health in Glasgow deteriorates, although this appears to be mediated by the migration route and status, since this affects both where migrants live in the city, in what concentrations and with what support mechanisms, either self-provided or provided by state and voluntary agencies. Thus, our study shows that the healthy immigrant effect is a product of, and differentiated by, both geographical and institutional factors.

## Footnotes

1 A person given refugee status is normally granted leave to remain in the UK for 5 years, and at the end of that period can apply for indefinite leave to remain. Source: http://www.refugeecouncil.org.uk/glossary#R.

2 School leaving certificate (slc) was used to indicate the successful completion of compulsory schooling without necessarily passing exams, though we cannot be certain of its status in other countries. The vast majority in the first category on the binary education variable had no qualifications rather than slc. A list of other qualifications were offered to respondents based on UK provision, although the list included more generic descriptors such as ‘trade qualification’, ‘business qualification’, ‘first degree’ and ‘higher degree’.
